# Blood flow on ultrasound imaging is a predictor of lump margin status in breast-conserving patients: a retrospective matching study

**DOI:** 10.1186/s40001-023-01356-4

**Published:** 2023-09-20

**Authors:** Rong Zhao, Jianyong Zhang, Jinnan Gao

**Affiliations:** 1 General Surgery Department, Shanxi Bethune Hospital, Shanxi Academy of Medical Sciences, Tongji Shanxi Hospital, Third Hospital of Shanxi Medical University, Taiyuan, 030032 China; 2 Third Hospital of Shanxi Medical University, Shanxi Bethune Hospital, Shanxi Academy of Medical Sciences, Tongji Shanxi Hospital, Taiyuan, 030032 China

**Keywords:** Breast cancer, Blood flow, Breast-conserving surgery, Predict, Ultrasonography

## Abstract

**Purpose:**

This study investigated the relationship between breast ultrasound features and lump margin status in breast-conserving patients.

**Methods:**

A single-institution database and medical records system were searched to identify patients who had undergone breast-conserving surgery between 2015 and 2022. Patients were divided into case and control groups based on their postoperative margin status, and different matching methods [case–control matching (CCM) and propensity score matching (PSM)] were used to match the cases and controls at a ratio of 1:1.

**Results:**

Before matching, patients with positive margins were more likely to have a tumor with increased blood flow (OR = 2.90, 95% CI 1.83–4.61, *p* < 0.001) and microcalcifications (OR = 2.22, 95% CI 1.44–3.42, *p* < 0.001). Among the 83 pairs of CCM subjects, patients with positive margins were prone to increased blood flow (*p* = 0.007) and crab sign (*p* = 0.040). In addition, there was a significant difference in blood flow (*p* = 0.030) among PSM subjects. After adjusting for the unbalanced factors, the same results were obtained.

**Conclusions:**

Ultrasound blood flow significantly predicts the status of breast-conserving margins, but further studies are required to verify our findings.

## Background

Breast-conserving surgery (BCS) is the standard therapy for women with early-stage breast cancer [1]. Clean surgical margins in BCS provide superior protection against recurrence [2], with postoperative pathological results serving as the gold standard for margin status diagnosis. Further surgery is required if the margin is positive and the rates in literature are elevated to 20–40% [3–6]. Positive margins are problematic, and in addition to economic and aesthetic factors, they place patients under tremendous psychological stress. After one failed attempt at breast-conserving surgery, some women will opt for mastectomy.

Intraoperative analysis of margins could lower rates of further surgery [3]. For example, the reoperation rate decreased to approximately 15% after adopting the fresh frozen section method (FSM) [7, 8]; however, FSM is complicated, time-consuming, and places a heavy burden on pathologists [9, 10]. If frozen margins are positive, they are re-excised, and the new margin must be evaluated by intraoperative frozen section analysis; thus, this method has not been widely adopted [11].

Some studies [12–17] explored this topic and identified several factors relevant to margin status, such as age, BMI, tumor size, multifocal disease, the presence of extensive intraductal component (EIC), microcalcifications in mammography, and lymph node stage. Ultrasound features of malignancy have been rarely mentioned, but breast ultrasound is a routine evaluation before BCS [18], thus will impose no extra economic burden. We hypothesized that there are unexplored underlying indicators on ultrasound that could contribute to predicting breast-conserving margin status. Therefore, this study investigated whether ultrasound features can predict margin status in patients who have undergone breast-conserving surgery.

## Methods

### Study population

With Institutional Review Board approval, we identified all patients with invasive breast cancer confirmed by core needle biopsy who had undergone breast-conserving surgery at Shanxi Bethune Hospital between 1 January 2015 and 31 June 2022 (all were of Han Chinese descent). Patients who had neoadjuvant therapy and the biopsy tissue presented intraductal components or lobular histology or absence of margin status were excluded. The case group included patients with positive margins, and the control group included other patients. Bilateral BCS was recorded as two independent cases.

### Data collection

Pathological and demographic information was obtained from the hospital database. Clinical examination (palpability), location, and imaging features were retrieved from the electronic medical records. Imaging features were collected from ultrasonography, mammography, and breast magnetic resonance imaging (MRI) reports routinely performed before breast-conserving surgery.

Mammography provides information about microcalcifications, and because MRI is a highly sensitive examination, it is mainly used for evaluating multiple lesions. Multiple lesions included multifocal (multiple areas of tumor in one quadrant) and multicentric (multiple areas of tumor affecting more than one quadrant) [19] and were confirmed pathologically. If breast magnetic resonance imaging is considered to involve multiple lesions and is subsequently confirmed by postoperative pathology, it is categorized as the "multiple lesions" group. If breast magnetic resonance imaging is considered to involve multiple lesions, but postoperative pathology confirms some of these lesions to be benign, then the patient is classified as the "single lesion" group.

**Fig. 1:**
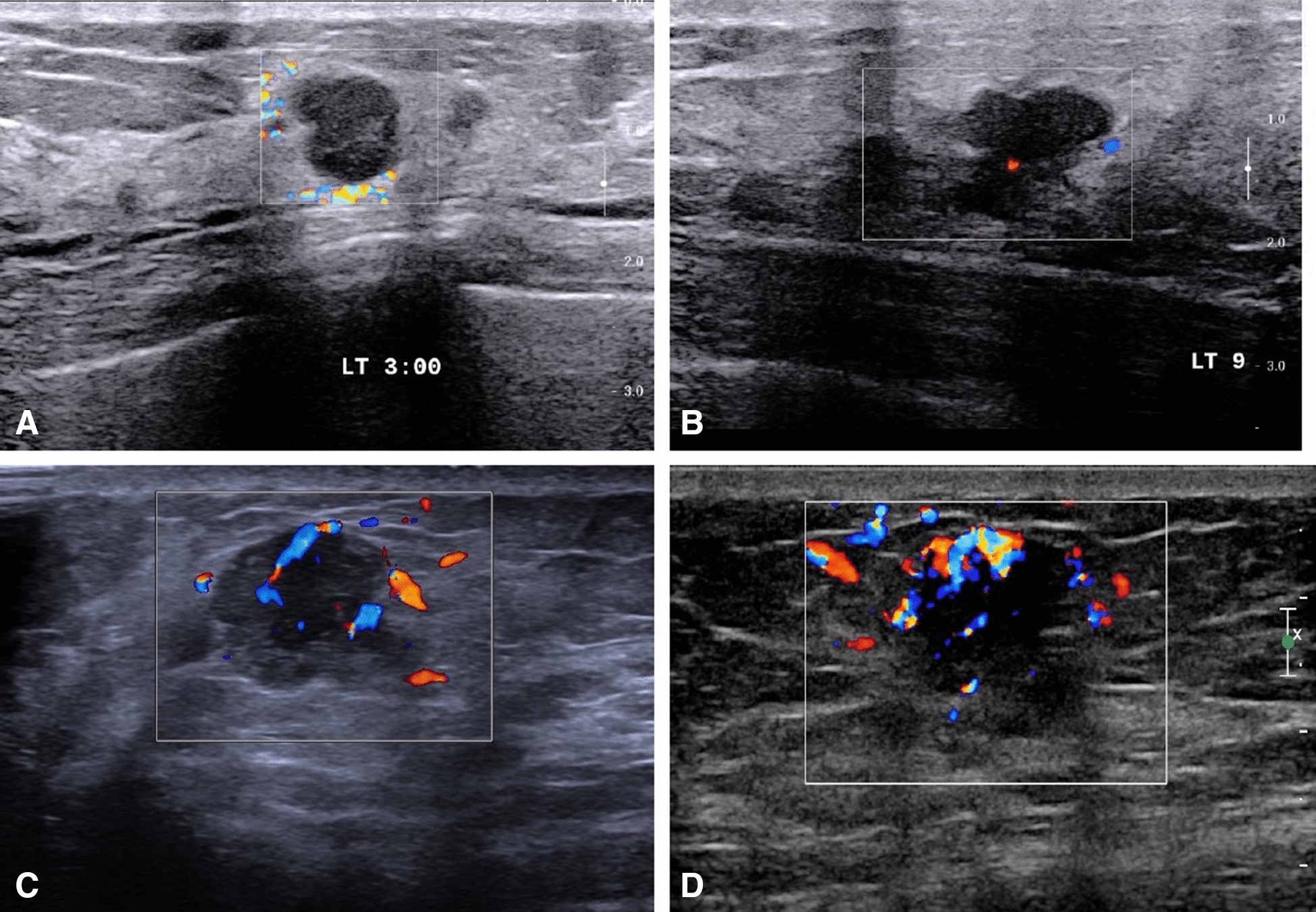
Schematic diagram for the four blood flow signal grades [no flow (Level 0): **A**, minimal flow (Level I): **B**, moderate flow (Level II): **C**, marked flow (Level III): **D**]

Ultrasound is useful for lump edge assessment, which is the borderline between tumor and normal tissue, and blood flow assessment. Typically, the tumor margins are not smooth and classified into three types: arcade-like structure, crab sign, and spiculated margin. A single lesion could present three manifestations at the same time. Referring to the Adler grading criteria [20], blood flow can be divided into four levels. Level 0, no blood flow signals detected within the mass. Level I, slight blood flow present, with 1 to 2 punctate or slender rod-like tumor vessels visible and rod-like blood flow does not exceed half the diameter of the lesion. Level II, moderate blood flow present, with 3 to 4 punctate vessels visible or a longer vessel entering the lesion; the length of the vessel may be close to or exceed the radius of the mass. Level III, abundant blood flow present, with ≥ 5 punctate vessels visible or 2 longer vessels visible. The schematic diagram for the four blood flow signal grades can be found in Fig. 1. In this study, "increased flow" refers to levels II and III. Crab sign, spiculated margin, and increased blood flow were recorded for those factors which may increase the possibility of a positive margin. Two sonographers (at least one with > 10 years of experience) independently issued the ultrasonography report. If there were discrepancies, they were adjudicated by a third sonographer. Ultrasound examinations were performed using a Philips iU22 equipped with an L12–5 probe (Philips Medical Systems, Bothel, WA).

### Surgical procedure and the pathological assessment

Each surgery is performed by an experienced breast surgeon. The primary surgeon determines the incision location, the use of localization techniques, and other operative details based on factors, such as the tumor's position, size, and the condition of the breast.

It is standard practice in our institution that all breast cancer lumps are evaluated by frozen section examination. When the tissue is received in the laboratory, it is "annotated" to accurately represent the in vivo position. Sections for margin evaluation are perpendicular to the inked surface, and the distance between carcinoma and the inked margin can be measured microscopically. Two breast pathologists with > 10 years of experience independently assessed the specimens. If the margins are positive, they are usually re-excised, and the new margin is evaluated again by intraoperative frozen section analysis. All specimens are paraffin-embedded and tested after BCS. Reoperation is recommended if the permanent margin is unclear (except for the positive frozen margin with re-excision). In our study, the margin result was based on the postoperative pathological result. If intraoperative re-excision was performed, the slide of the primary excision was used as the final result. The positive definition is consistent with current guidelines [21, 22]: no inked margins for invasive cancer.

### Subject matching methods

Matching ensures that the distributions of confounding variables are as close to identical as possible. CCM (case–control matching) involves selecting subjects according to the matching factors, and subjects in the control group are completely consistent with subjects in the case group [23]. In PSM (propensity score matching), the control selection for each case is based on the propensity score rather than matching factors [24]. To balance the baseline characteristics between groups, CCM and PSM were used to match patients with positive and negative margins at a 1:1 ratio. The matching parameters included age, BMI, menopausal status, tumor location, palpability, multiple lesions, tumor size, and microcalcifications. In CCM, the maximum allowance of difference (MAD) of BMI was set to 1 kg/m^2^, the MAD of the tumor size was 1.0 cm, and an exact match was used for age, menopausal status, tumor location, palpability, multiple lesions, and microcalcifications. In PSM, the matching tolerance score between the case group and the control group was set at 0.02.

### Statistical analysis

Normality was tested with the Kolmogorov–Smirnov test for continuous variables, which were expressed as mean ± standard deviation (SD) and analyzed by analysis of variance (ANOVA). Categorical variables were expressed as a proportion, and the paired chi-square test (χ^2^) was used to compare differences between groups. Missing values were handled using multiple imputations. All statistics were performed in SPSS 26.0, and all tests were two-sided. A *P* value < 0.05 was considered statistically significant. The standardized mean difference (SMD) was calculated to assess the balance after matching, and the threshold was set for a mean difference of 0.1 [25]. To control confounding variables, conditional logistic regression models were built and used to estimate the odds ratios (OR) and their associated 95% confidence intervals (CIs).

## Results

### Total population analysis

#### Univariate analyses

**Fig. 2:**
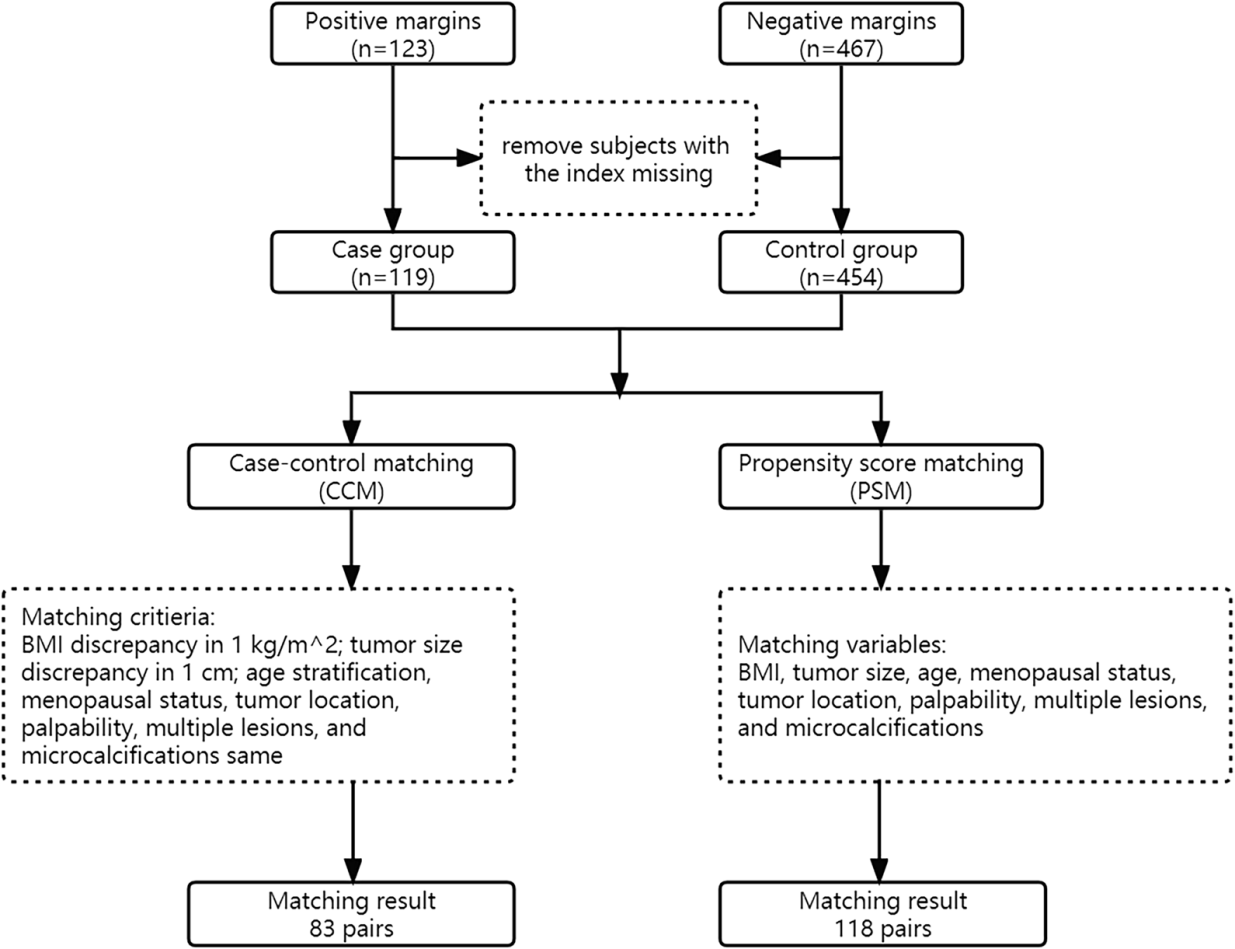
Flow chart

In total, 630 consecutive patients who underwent BCS from 2015 through 2022 were identified, of which 573 patients met the inclusion criteria (Fig. 2). There were 119 patients in the case group and 454 in the control group. Baseline demographics, clinical and imaging characteristics in the total population are summarized in Table 1. Microcalcifications in mammography and blood flow in ultrasound were significantly different between the case and control groups (*p* < 0.05).

**Table 1:** Comparison of baseline characteristics between the two groups in total populations

	Positive margins	Negative margins	*P*
*N*	119	454	
Age (years)			0.421
≤ 50	53 (44.5%)	221 (48.7%)	
> 50	66 (55.5%)	233 (51.3%)	
BMI	24.9 ± 3.3	25.0 ± 3.5	0.770
Menopausal status			0.955
Postmenopausal	56 (47.1%)	210 (46.8%)	
Non-postmenopausal	63 (52.9%)	239 (53.2%)	
Tumor location			0.871
Upper outer quadrant	49 (41.2%)	182 (40.4%)	
The other quadrants	70 (58.8%)	269 (59.6%)	
Palpability			0.156
Yes	104 (87.4%)	416 (91.6%)	
No	15 (12.6%)	38 (8.4%)	
Multiple lesions			0.646
Yes	9 (7.6%)	29 (6.4%)	
No	110 (92.4%)	425 (93.6%)	
Tumor size (cm)	2.0 ± 0.9	2.0 ± 0.8	0.440
Microcalcifications			0.001
Yes	51 (42.9%)	121 (26.7%)	
No	68 (57.1%)	333 (73.3%)	
*Ultrasonic features*			
Increased blood flow			0.000
Yes	41 (34.5%)	74 (16.3%)	
No	78 (65.5%)	380 (83.7%)	
Crab sign			0.557
Yes	36 (30.3%)	125 (27.5%)	
No	83 (69.7%)	329 (72.5%)	
Spiculated margin			0.826
Yes	43 (36.1%)	169 (37.2%)	
No	76 (63.9%)	285 (62.8%)	

*BMI* Body Mass Index

#### Multivariate analyses

To remove the influence of microcalcifications, a logistic model including microcalcifications and ultrasonic features was built, indicating that the tumor with microcalcifications (OR = 2.22, 95% CI 1.44–3.42, *p* < 0.001) and increased blood flow (OR = 2.90, 95% CI 1.83–4.61, *p* < 0.001) was more likely to be margin-positive (Table 2).

**Table 2:** Comparison of baseline characteristics between the two groups in total populations

	OR	95% CI	*P*
Microcalcifications (yes vs. no)	2.22	1.44–3.42	0.000
Increased blood flow (yes vs. no)	2.90	1.83–4.61	0.000
Crab sign (yes vs. no)	0.80	0.49–1.30	0.366
Spiculated margin (yes vs. no)	1.05	0.66–1.69	0.826

### Comparisons after PS and CC matching

#### Univariate analyses

Among the 83 case–control matched pairs of subjects, the p values of all variables (except for ultrasonic features) between the two groups were greater than 0.05. However, the SMD of tumor size was 22.6%, greater than 10% (Table 3). The same results were also found for the 118 pairs of PSM subjects (Table 4). Variables with an SMD > 10% were multiple lesions (18.5%) and tumor size (14.6%). Univariate analyses revealed that tumors with positive margins were prone to increased blood flow (*p* = 0.007) and crab sign (*p* = 0.040) among the CCM subjects, with only a significant difference in blood flow for the PSM subjects (p = 0.030) (Table 5).

**Table 3:** Comparison of baseline characteristics between the two groups after case–control matching

	Positive margins	Negative margins	P	SMD (%)
*N*	83	83		
Age (years)			1.000	0.0
≤ 50	40 (48.2%)	40 (48.2%)		
> 50	43 (51.8%)	43 (51.8%)		
BMI	25.1 ± 3.4	25.2 ± 3.4	0.913	1.7
Menopausal status			1.000	0.0
Postmenopausal	41 (49.4%)	41 (49.4%)		
Non-postmenopausal	42 (50.6%)	42 (50.6%)		
Tumor location			1.000	0.0
Upper outer quadrant	32 (38.6%)	32 (38.6%)		
The other quadrants	51 (61.4%)	51 (61.4%)		
Palpability			1.000	0.0
Yes	78 (94.0%)	78 (94.0%)		
No	5 (6.0%)	5 (6.0%)		
Multiple lesions			1.000	0.0
Yes	1 (1.2%)	1 (1.2%)		
No	82 (98.8)	82 (98.8)		
Tumor size (cm)	2.0 ± 0.73	1.9 ± 0.64	0.190	22.6
Microcalcifications			1.000	0.0
Yes	26 (31.3%)	26 (31.3%)		
No	57 (68.7%)	57 (68.7%)		

*BMI* Body Mass Index, *SMD* standardized mean difference

**Table 4:** Comparison of baseline characteristics between the two groups after propensity score matching

	Positive margins	Negative margins	*P*	SMD(%)
*N*	118	118		
Age (years)			0.896	1.6
≤ 50	53 (44.9%)	52 (44.1%)		
> 50	65 (55.1%)	66 (55.9%)		
BMI	24.9 ± 3.4	25.3 ± 3.7	0.445	9.9
Menopausal status			0.794	3.4
Postmenopausal	56 (47.5%)	58 (49.2%)		
Non-postmenopausal	62 (52.5%)	60 (50.8%)		
Tumor location			0.598	6.9
Upper outer quadrant	48 (40.7%)	52 (44.1%)		
The other quadrants	70 (59.3%)	66 (55.9%)		
Palpability			1.000	0.0
Yes	103 (87.3%)	103 (87.3%)		
No	15 (12.7%)	15 (12.7%)		
Multiple lesions			0.154	18.5
Yes	9 (7.6%)	4 (3.4%)		
No	109 (92.4%)	114 (96.6%)		
Tumor size (cm)	2.0 ± 0.9	2.1 ± 1.0	0.263	14.6
Microcalcifications			0.596	6.9
Yes	50 (42.4%)	46 (39.0%)		
No	68 (57.6%)	72 (61.0%)		

*BMI* Body Mass Index, *SMD* standardized mean difference

**Table 5:** Differences of ultrasonic features between the two groups

	CCM			PSM		
	Positive	Negative	*P*	Positive	Negative	*P*
Increased blood flow			0.017			0.030
Yes	31 (37.3%)	17 (20.5%)		41 (34.7%)	26 (22.0%)	
No	52 (62.7%)	66 (79.5%)		77 (65.3%)	92 (78.0%)	
Crab sign			0.040			0.384
Yes	30 (36.1%)	18 (21.7%)		36 (30.5%)	30 (25.4%)	
No	53 (63.9%)	65 (78.3%)		82 (69.5%)	88 (74.6%)	
Spiculated margin			0.748			0.788
Yes	32 (38.6%)	30 (36.1%)		43 (36.4%)	45 (38.1%)	
No	51 (61.4%)	53 (63.9%)		75 (63.6%)	73 (61.9%)	

*CCM* case–control matching, *PSM* propensity score matching

#### Multivariate analysis

To remove the influence of unbalanced variables (SMD > 10%), logistic models were built, showing that for CCM and PSM subjects, the tumor with positive margins tended to have increased blood flow (OR = 2.51, 95% CI 1.21–5.19, *p* = 0.013 in CCM and OR = 2.14, 95% CI 1.18–3.89, *p* = 0.012 in PSM). The crab sign was only significantly different between groups for the CCM subjects (OR = 2.48, 95% CI 1.15–5.35, *p* = 0.021), and there was no significant difference in spiculated margin between the groups for both CCM and PSM subjects (Table 6).

**Table 6:** Results of the logistics model analysis

	CCM		PSM	
	OR (95% CI)	*P*	OR (95% CI)	*P*
Multiple lesions (yes vs. no)	–		2.56 (0.75–8.82)	0.136
Tumor size (cm)	0.81 (0.50–1.31)	0.396	1.22 (0.92–1.63)	0.175
Increased blood flow (yes vs. no)	2.51 (1.21–5.19)	0.013	2.14 (1.18–3.89)	0.012
Crab sign (yes vs. no)	2.48 (1.15–5.35)	0.021	1.52 (0.81–2.85)	0.189
Spiculated margin (yes vs. no)	0.72 (0.35–1.48)	0.370	0.83 (0.46–1.48)	0.520

*CCM* case–control matching, *PSM* propensity score matching

## Discussion

Previous studies on breast ultrasound and margin status have primarily focused on the use of intraoperative ultrasound, which can reduce the rate of positive margins in breast-conserving surgery [4, 26, 27]. In recent years, with the rapid advancement of information technology, some scholars have started to explore the impact of computer-assisted ultrasound assessment on margin status [28, 29]. We thoroughly reviewed the literature and did not find any scholars investigating the relationship between the basic ultrasound characteristics of preoperative breast masses and margin status in breast-conserving surgery. Therefore, our study, in a sense, holds pioneering significance.

The present study indicated that increased blood flow predicts positive margins. This may be because angiogenesis is essential for tumor growth and metastasis, and neovascularization of the tumor vessels is characterized by a more rigid muscle structure, increased vessel wall permeability, and arteriovenous short circuits that provide increased blood flow to the lesion [30, 31]. Therefore, increased blood flow may be linked to smaller satellite foci that are difficult to identify without a microscope. We believe this finding is pragmatic, because ultrasound is a routine and cost-effective examination before BCS. In addition, the few models that have been developed to predict breast-conserving margin status have been externally validated [12, 32, 33]. This newly discovered indicator, blood flow, can be integrated into margin predictive models, and the model generalization may be optimized.

It is noteworthy that blood flow is inevitably subjective and dependent on accurate reporting by the sonographers. However, the ultrasonography report was determined by at least two sonographers in our institution and was classified as the lesion area without flow, with similar flow to the perirhinal area, and with increased flow [34]; therefore, the extent of subjectivity is acceptable.

The positive margin definition in breast-conserving surgery is controversial [35]. The Society of Surgical Oncology (SSO), the American Society for Radiation Oncology (ASTRO), and the American Society of Clinical Oncology (ASCO) released a consensus statement in 2014 based on the available data, and the updated definition is less strict than the previous version [22, 36]. This criterion (no inked margins for invasive cancer) was adopted in our study, and this may be one of the potential reasons for the low rate of positive margins (20.7%) in our study compared to other reports [4, 10]. A recently published meta-analysis supported this conjecture (from 22% to 14%; OR = 0.65; 95% CI 0.54–0.78; *p* < 0.0001) [37].

Previous studies have been committed to certain features to improve the prediction power of the margin status [38]. Microcalcifications are a significant positive predictor in numerous previous studies [12–15, 17]; possibly because they are a major manifestation of ductal carcinoma in situ (DCIS), and disseminated lesions easily develop along the ducts. Our results align with the former research, but microcalcifications were regarded as the main confounding factor in the matching process, because our main study purpose was to explore the relationship between ultrasonic features and margin status.

Other factors, such as extensive intraductal component, lymph node stage, grade, and immunohistochemistry results, had predictive potential in some studies [12, 14, 32]; however, those factors were not gathered in our investigation. Previous reports [39, 40] have indicated that the false-negative finding may be elevated to 32% and 46% relating to EIC in the puncture tissues. The clinic status of lymph nodes before surgery was not collected for the same reason. Ultrasound has been widely used to preoperatively determine ALN status [41], but the diagnostic performance of axillary ultrasound was poor with an area under the receiver operating characteristic curve (AUC) of 0.585–0.719 [42]. In some institutions, grade and immunohistochemistry results are not routinely available. To enable broad generalizability and accuracy of results, we decided not to incorporate those factors into our study.

There are several limitations to the study. First, we acknowledge that in studies involving surgical interventions, the influence of the operating surgeon on the research outcomes is unquestionable. However, regrettably, our study was unable to incorporate this crucial factor. Although we could access the responsible surgeon for each surgery through our medical record system, the actual operating surgeon might not have been accurately documented and could potentially have been an assistant instead (a situation prevalent in our medical institution). Adhering to rigorous principles, we ultimately chose not to include the factor of the operating surgeon. Second, breast density was a potential predictor in previous studies [14, 15], but we did not have relevant data, so breast density was not matched in our study. Third, this is a single-center study, so more multicenter data are needed to validate our findings. Fourth, the sample size was relatively small, so the study may not have been adequately powered to detect some associations between blood flow and positive margin status.

## Conclusion

The present study confirmed blood flow as a potential predictor of positive margin status. Wider excision could be considered before BCS if there is increased blood flow in preoperative evaluation, thus facilitating clinical decision-making.

## Data Availability

The data originates from personal data and can, therefore, not be publicly available. The datasets used and/or analyzed during the current study are available from the corresponding author upon reasonable request.

## Abbreviations

CCM: Case–control matching
PSM: Propensity score matching
BCS: Breast-conserving surgery
FSM: Frozen section method
EIC: Extensive intraductal component
DCIS: Ductal carcinoma in situ
MRI: Magnetic resonance imaging
MAD: Maximum allowance of difference
BMI: Body mass index
SD: Standard deviation
ANOVA: Analysis of variance
SMD: The standardized mean difference
OR: Odds ratios
CIs: Confidence intervals
AUC: Area under the receiver operating characteristic curve
